# Effect of gum chewing and cold therapy on postoperative cesarean women’s self-assessed pain levels and narcotics use: a comparative study

**DOI:** 10.1186/s12884-025-07770-2

**Published:** 2025-06-07

**Authors:** Reda Mhmoud Mohamed Hables, Nor El-Hoda Mohamed El-Sayed ElShabory, Esraa Mostafa Abd El-Aty Ibrahim

**Affiliations:** 1 Assistant Professor of Obstetrics and Gynecological Nursing, Faculty of Nursing, Alexandria University, Alexandria, Egypt; 2 Assistant Professor of Maternity, Gynecology and Obstetrics Nursing, Faculty of Nursing, Port-Said University, Port-Said, Egypt

**Keywords:** Cesarean section, Cold therapy, Gum chewing, Narcotic use, Pain

## Abstract

**Background:**

One of the most important things for women having cesarean deliveries to focus on is effective pain management. Nursing practice to lessen labor pain may be significantly impacted by the application of a nonpharmacologic treatment.

**Aim:**

Evaluate the effect of gum chewing and cold therapy on postoperative cesarean women’s self-assessed pain levels and narcotics use.

**Method:**

A Quasi Experimental intervention design was used in the study at inpatient units of Dar Sahet Elmar`Aa hospital that follow Egypt healthcare authority in Port Said city. Purposive sample of 159 pregnant women who enrolled randomly in the three parallel groups. Two tools were used for collecting data in the study as A self-Administered questionnaire and the visual analog scale (VAS) were used.

**Results:**

The overall mean VAS score among the Cold pack gel group was 4.216 ± 0.716, Gum chewing group was 4.314 ± 0.908, while control group was 5.714 ± 1.232 with statistically significance differences among groups. Furthermore, over one quarter in cold therapy group, less than one quarter in Gum chewing group (32.1 and 28.3%, respectively) stopped taking analgesics 24 h after surgery, compared to only 7.5% of control group.

**Conclusion:**

Cold pack gel and gum chewing offer simple and cost-effective alternatives for postoperative pain management; however, cold pack gel has been shown to be substantially more effective in improving pain scores at four, eight, twelve, and twenty-four hours after surgery. In addition to its impact on reducing the need for opioid prescription drugs.

Trial Registration Number (TRN).

The study protocol was registered by the Research Ethics Committee of the Faculty of Nursing, Port Said University with code number: NUR22 on 6/2/2023.

## Background

One of the most popular surgical procedures is the cesarean section (CS). Over the world, the cesarean rate has skyrocketed, surpassing 30% in some countries. According to the latest statistics gathered from 150 countries, 18.6% of all births occur through cesarean sections, with percentages ranging from 6% to 27.2% in those with the smallest and the most developed regions, respectively, with an average yearly growth rate of 4.4% [1].

The number of C-sections performed in Egypt has considerably grown. In Egypt these days, one in six deliveries are thought to include a C-section. According to data from the Central Agency for Public Mobilization and Statistics (CAPMAS) [2], it represented 72% of deliveries via C-section in 2021, up from 52% in 2014, when it ranked first globally. Also, data from the World Health Organization (WHO) indicate that the risk of maternal death related with cesarean delivery is three to seven times higher than that of vaginal delivery [3].

One of the main issues with surgery, especially CS, is pain. The number of CS cases has dramatically increased during the last 20 years, making it the most common surgical procedure performed globally. In the first 48 h following surgery, a caesarean section typically causes moderate to severe pain [4]. Many patients still have moderate to severe postoperative pain following a caesarean section, despite advancements in our understanding of the biology of post-surgery pain and the introduction of new analgesics and delivery procedures [5].

Pain following surgery is often nociceptive, meaning that it results from lesions in the tissues or organs that cause nociceptive stimuli to be felt as painful. Neuropathic pain can also occur in the event of a direct nerve injury, as well as in cases of strain or compression. Even though postoperative pain is a physiologic occurrence, there may be more dangers to people's health if this pain is not properly relieved [6]. Along with detrimental impacts on the central nervous system, the adverse consequences also include neuroendocrine changes involving the hypophysis and adrenal gland responses. These changes can have an adverse influence on several organic systems, including the gastrointestinal, respiratory, and cardiovascular systems. Chronic pain is also predicted by very intense surgical pain [7].

Furthermore, it is challenging to measure pain scientifically because it is a subjective feeling. Since measuring pain necessitates converting a subjective attribute into an objective one, the widely used pain scores might not accurately represent the patient's level of suffering. To ensure accuracy in the pain evaluation, it is necessary to align it with the woman's preferences about the mode and time of labor [8]. Despite this, the Visual Analogue Scale (VAS) remains a valuable instrument for the statistical analysis of pain. When evaluating pain relief as well as pain intensity, the VAS is helpful. One of the pain rating instruments that Hayes and Patterson employed for the first time was VAS [9]. According to [10], scores are indicated by handwriting a mark on a line of 10 cm, which represents a continuum ranging from "no pain" to "worst pain."

For women having cesarean deliveries, efficient pain management need to be a top concern. Inadequate management of pain during surgery has been linked to a number of problems, including increased opioid usage, postpartum depression, delayed functional recovery, and problems with maternal–fetal bonding. The foundational idea of pain management following cesarean delivery is multimodal analgesia [11]. Unless there is a contraindication, it is advised that all women having a cesarean delivery under neuraxial anesthesia utilize neuraxial morphine and opioid-sparing supplements like acetaminophen and scheduled nonsteroidal anti-inflammatory drugs [12].

Inadequate management of postoperative pain can considerably increase surgical patients' morbidity, delaying their recuperation and return to normal functional activities. For a patient whose expectation is to care for her newborn soon after surgery, prompt recovery is extremely crucial. Several factors that may impact pain following cesarean section have been documented in the literature [13]; medication requirements; religious and spiritual beliefs; pain threshold; and anxiety [14].

One approach to lessen discomfort is to use cold therapy. Compared to analgesic medicines, it is less expensive and has less adverse effects. Vasoconstriction is the result of physiological processes that lower tissue blood flow. According to [15], it lowers inflammation, oxygen requirements, muscle spasm, and tissue metabolism. By reducing the activation threshold of tissue nociceptors and the conduction velocity of nerve impulses, hypothermic effects at the local location can communicate with the spinal cord through neurologic and vascular pathways. For this reason, orthopedic, general, and gynecologic surgeries employ cold therapy as an extra postoperative pain management technique [16].

Another non-pharmacological approach to reduce pain and anxiety is to chew gum. It is easy to use, affordable, practical, noninvasive, and patient-friendly. Chewing gum is a similar pain reliever as analgesics, according to a number of earlier research. While only one study indicated that chewing gum relieves pain more effectively than prescription medications [17].

Promoting nonpharmacological methods, like as cold therapy and gum chewing, is essential for a nurse to help patients with postpartum pain issues following cesarean surgery. Before enabling the patient to chew gum, be sure they can swallow and are cognizant. In addition, monitoring the mother's response to the therapies in order to minimize any negative effects and adjusting the treatment plan as needed [18].

### Significant of the study

The number of C-sections performed in Egypt has dramatically grown. In Egypt these days, one in every six deliveries is thought to have a C-section. In terms of the total number of C-section births, it represents 72% in 2021, up from 52% in 2014, when the Central Agency for Public Mobilization and Statistics held the top spot worldwide [2].

Pain following a cesarean section is seen as a serious issue. Insufficient management of postoperative pain can have a substantial impact on women's morbidity, delaying their recuperation and capacity to resume their regular activities. This, in turn, is linked to a higher prevalence of severe pain [19].

Nowadays, there is a growing interest in non-pharmacologic treatments due to their noninvasive nature and lack of severe side effects. Several prior studies have suggested that giving cold therapy or chewing gum may speed up a woman's recovery from anesthesia complications following cesarean delivery. By giving these nursing instructions, nurses can accelerate the mother's healing process and help her adjust to a new life and role, which will benefit the nation's economy [20]. However, no prior research in Port Said city has compared the effects of gum chewing and cold therapy on postoperative cesarean women’s self-assessed pain levels and narcotics use. This study was conducted to address this gap.

### Aim of the study

This study aimed to evaluate the effect of gum chewing and cold therapy on postoperative cesarean women’s self-assessed pain levels and narcotics use.

### Research hypothesis

- H_0_: Gum chewing, and cold therapy decrease the postoperative cesarean women’s self-assessed pain levels with no side effects.
- H_1:_ Gum chewing, and cold therapy decrease women dependent on narcotics use postoperative cesarean.

## Method

### Research design

A Quasi Experimental intervention study involved three groups (gum chewing group, cold pack gel group and control group) was used to conduct this study.

### Study setting

This study was carried out in inpatient units of Dar Sahet Elmar`Aa hospital that follow Egypt healthcare authority in Port Said city.

### Subjects

Expectant mothers who had cesarean sections performed under spinal anesthesia were included in the Purposive sample. In all three parallel groups, the women under study were enrolled equally and at random.

- Group A, exposed to gum chewing sugar free.
- Group B, exposed to cold pack gel.
- Group C, The control group received standard routine postoperative care.

### Sample size

The study by [21] was used to establish the sample size for this investigation. We applied the formula for the test of difference in two proportions of independence. At 0.01 and 0.05, respectively, the alpha and beta were set. the formula used to calculate the sample size for testing the difference is as follows:

$$n\;=\;\frac{\left(Z_{a/2}+\;Z_\beta\right)^2\;\left(k\;\sigma^2\right)}{d^2}$$

The sample size was 48 participants, which was obtained from the calculations. An additional 10% was added to avoid data loss, making the sample size 53 participants per one group. The total number of expectant mothers was 159 across the three groups. The total samples were then randomly distributed among the three groups under study with Random Number Generator Allocation via Excel.

The sample was selected using the inclusion criteria listed below: Participants: Following the cesarean surgery, women were cognizant, in good condition, and there had been no major problems (diabetes mellitus, severe eclampsia, gestational hypertension, etc.).

### Tools of data collection

#### Tool I: a self-administered questionnaire

It has been adapted from [22] into English. After making changes, the researcher translated it from English into Arabic language. There are three primary parts to it.

- 1^st^. part: General characteristics: Age, occupation, height, weight, BMI, and chronic illness are all included.
- 2^nd^ part: Medical history: Parity, prior cesarean birth, pregnancy problems, indications for C/S, estimated blood loss, operating time, incision wound length, and duration of hospital stay are all included.
- 3^rd^ part: During the first 24 hours following a cesarean section, pain management options include diclofenac alone, pethidine alone, intrathecal morphine solely, using various analgesics, or using none at all.

#### Tool II: Visual analogue scale (VAS)

**Fig. 1:**
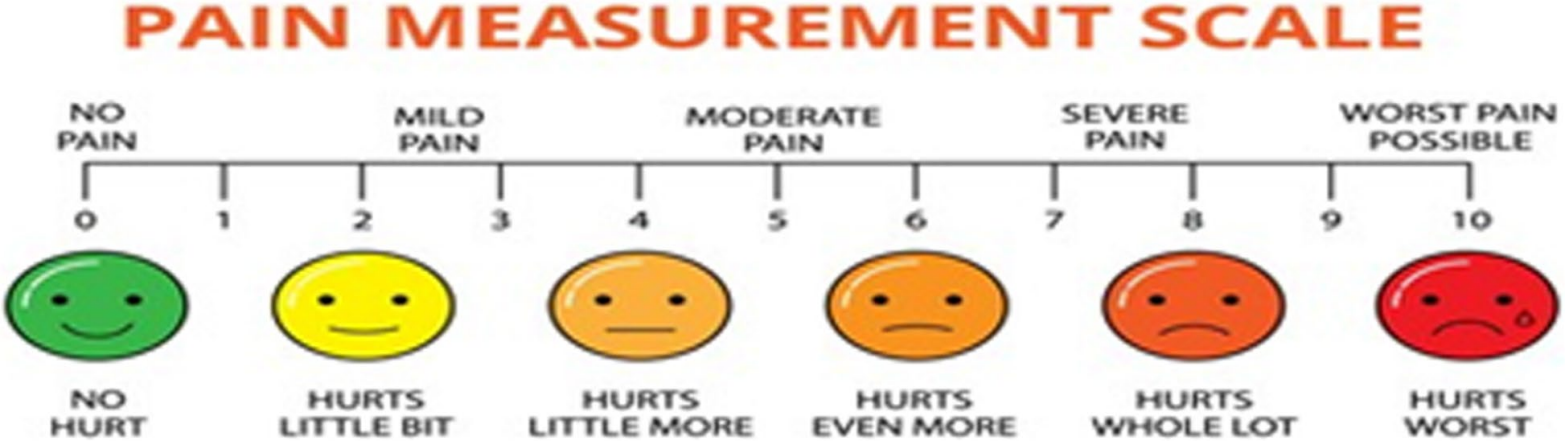
A visual analog scale that has been used in clinical settings

The researcher translated it from English to Arabic language after adopting it from [10]. When evaluating pain, participants who fulfilled the study's requirements used the visual analogue scale (VAS), which runs from 0 to 10, with 10 being the highest level of pain (Fig. 1).

The woman self-completed the pain VAS by marking the spot on the VAS line that represented the degree of her pain with a line perpendicular to the line. The distance between the patient's mark and the no pain anchor on the 10 lines was measured with a foot ruler to calculate the pain score, which ranged from 0 to 10. The pain scale was set at 0 for no pain, 1–3 for mild pain, 4–6 for moderate pain, and 7–10 for severe pain. The patient's pain was measured as soon as they entered the recovery area (0 h), 4 h, 8 h, 12 h, and 24 h later in the postoperatively obstetric ward.

### Tool content validity

Five experts in obstetrics, and gynecology from the medical and nursing faculties at Port-Said University reviewed the data collection instruments that the researcher had developed. The test's objective was to evaluate the tools' applicability, importance, scope, and clarity. After asking them for their opinions on the instrument's structure, organization, and coherence, the necessary changes were implemented.

### Content reliability

The reliability was confirmed through the computation of Cronbach's alpha coefficient. Its results of 0.86 for the visual analog scale (VAS) and 0.83 for a self-administrated questionnaire demonstrated high reliability.

### Ethical considerations

The ethics committee of the nursing faculty at Port Said University formally approved and recognized the conduct of the study under code NUR 6/2/2023(22). Furthermore, after obtaining the required clearances, the study was approved by the head manager of the previously mentioned sitting. Each woman included in the study's sample was informed of her option to take part or not. The researcher provided an explanation of the study's purpose to each of the female participants in the sample. They were told that all study data would be kept private and utilized only to forward the goals of the inquiry. The privacy of the subjects was always respected. Before being enrolled, the women filed written consent forms.

### Administrative design

The directors of the previously mentioned meeting in the city of Port Said received formal approval from the Directorate of Health and the Dean of the Nursing Faculty in order to obtain authorization prior to carrying out the study. They were given the option to decline participation and were reassured that the information they provided would only be used for research.

#### A pilot study

10% of the study's participants for the pilot research consisted of sixteen randomly selected women from the previously described sitting. This was done to assess the utility, clarity, and relevance of the created tool and to estimate the time required to finish the questionnaire. The questionnaire was altered, with some questions added and others deleted, especially in the areas that dealt with the habits and knowledge of female students. The pilot study's female participants were consequently excluded from the overall study population. There was no need for any adjustments.

### Field work

Data for the study were gathered from 15 September, 2023, to 31 December, 2023.

### Procedures

Procedures: There are three phases to the procedure: pre-intervention, intervention, and post-intervention.

### Pre intervention phase

After explaining the purpose of the study to each mother, the researcher got their signed agreement and gave them assurances regarding anonymity. In order to ascertain the type of group the lady was involved in, the researcher informed the mother that her group had been randomly assigned by selecting paper from a box that contained the three groups. Every participant was informed that she was free to leave the research at any time. After C/S, each participant was given a one-on-one interview in the recovery area to gather information about their clinical history, narcotic use, pain level, and sociodemographic characteristics. Four days a week, from 8 to 2 p.m., the researcher went to gather data.

#### Interventions phase

The researcher conducted the following intervention based on the previous randomly selected groups.

- Group A (Chewing sugar free gum group): The woman was instructed by the researcher to chew sugar-free Xylitol gum for 15 min after two hours following CS, and to repeat the process every hour after that.
- Group B (Cold pack gel group): received a tiny cloth bag with a cold pack gel. At -4°Celsius, the cold pack gel was maintained. After two hours following surgery, the cold pack gel was first applied over the surgical wound dressing. Every two hours, when the cold pack gel warmed up throughout a six-hour period, it was replaced.
- Group C (control group): got typical postoperative care routine. Intravenous fluids every 12 h and hospital protocol-followed care. Analgesia and intravenous fluids: Every woman in the hospital received the same intravenous fluids, which consisted of 1000 ml of 5% dextrose and regular saline, once every 12 h.It should be mentioned that when the woman is accustomed to feeding before 24 h and her bowel movements start early, these intravenous fluids should be stopped. The first IV fluid bottle the women were given in the recovery area contained voltaren injection (75 mg IV per 12 h), which was combined with the same standard analgesic medication for all of them.

### Post-intervention phase

After all study groups had received nursing instructions, the researcher looked into the vital signs and the timing of the return of bowel noises in each woman. The researcher measured the women's level of discomfort at 4, 8, 12, and 24 h following surgery using a visual analog scale. Additionally, evaluate how post-section pain is managed throughout the first 24 h. It took ten to fifteen minutes to complete this step.

### Statistical design

The Statistical Package for Social Sciences (SPSS) version 22 was used for statistical analysis and computerized data entry. Numbers, percentages, means, and standard deviations were among the analytical and descriptive statistics employed. One-way ANOVA on ranks, often known as the Kruskal–Wallis H test, is a non-parametric technique for determining if samples come from the same distribution. The comparison of two or more independent samples with the same or different sample sizes is what it's used for. A statistical test for categories of data is the chi-square (X2) test.

## Results

Table 1 displays the general characteristics of the three groups. There was statistically no difference in the general characteristics data between the two groups, which included age, height, weight, BMI, employment, and chronic illness. Nearly majority of the women in the study were free of chronic illnesses. Among them, housewives made up more than half.

**Table 1:** Distribution of studied groups according to their general characteristics (*N* = 53)

Items	Gum chewing (*N*=53)		Cold pack gel (*N*=53)		Control group (*N*=53)		*Kruskal Wallis p*.value
	N	%	N	%	N	%	
Age							
20 - <25	15	28.3	13	24.5	14	26.4	0.926 >0.05
25- <30	10	18.9	13	24.5	13	24.5	
30 – 35	10	18.9	11	20.8	10	18.9	
>35	18	33.9	16	30.2	16	30.2	
Mean±S.D	30.6±5.9		29.7±6.4		29.9±5.7		
Employment							*Chi-square*
Housewife	34	64.2	30	56.6	36	67.9	2.876 <0.05*
Self-employment	2	3.8	0	0	1	1.9	
Employee	17	32	23	43.4	16	30.2	
Weight							0.883 >0.05
Mean±S.D	69.51±6.7		70.15±8.9		69.74±7.12		
Height							1.120 >0.05
Mean±S.D	161.09±6.31		158.3±7.2		160±7.9		
BMI	27.13±4.2		26.97±3.6		27.01±3.00		1.067 >0.05
Mean±S.D							
Chronic disease							*Chi-square*
Yes	6	11.3	5	9.4	7	13.2	0.992
No	47	88.7	48	90.6	46	86.8	>0.05

Highly significant if *p* value <0.01

Significant if *p* value <0.05

Insignificant if *p* value >0.05

As shows in Table 2, the three groups' obstetrical characteristics were likewise similar. There were almost more than half of multipara women. No prior C/S delivery was made by about two thirds of them. For the most part, none of them had any pregnancy-related complications. Blood loss, operating time, incision wound, and hospital stay duration did not differ among the three groups under investigation (*p* value > 0.05). Indicators of C/S varied little amongst the three groups under study, with a *p*-value of less than 0.05.

**Table 2:** Number and percent distribution of studied groups according to their obstetrical characteristics (*N* = 53)

Items	Gum chewing (*N*=53)		Cold pack gel (*N*=53)		Control group (*N*=53)		*Chi-square* *p*. value
	N	%	N	%	N	%	
Parity							
Primipara	20	37.7	22	41.5	19	35.8	1.002
Multipara	33	62.3	31	58.5	34	64.2	>0.05
Previous cesarean delivery							
Yes	19	35.8	17	32.1	18	33.9	0.981
No	34	64.2	36	67.9	35	66.1	>0.05
Complications of pregnancy							
Yes	9	16.9	11	20.7	10	18.9	0.715
No	44	83.1	42	79.3	43	81.1	>0.05
Indications for C/S							
Previous C/S	16	30.2	14	26.4	13	24.5	3.618 <0.05*
Failure to progress of labor.	8	15.1	10	18.9	11	20.8	
Non-reassuring fetal status	8	15.1	13	24.5	14	26.4	
Breech presentation	11	20.8	10	18.9	8	15.1	
Other	10	18.8	6	11.3	7	13.2	
Estimate blood loss (ml) Mean±S.D	495.6± 147.1		507.8±164.0		523.1±129.8		*Kruskal Wallis* 1.223 >0.05
Operative time (min) Mean±S.D	46.52 ± 10.1		50.1± 9.13		48.11 ± 8.61		*Kruskal Wallis* 0.732 >0.05
Length of incisional wound Mean±S.D	10.92 ± 1.8		11.6± 1.3		10.25± 1.9		*Kruskal Wallis* 1.208 >0.05
Length of hospital stay (days)	1.4± 0.3		1.8±0.6		1.2±0.5		*Kruskal Wallis* 0.922 >0.05

Highly significant if *p* value <0.01

Significant if *p* value <0.05

Insignificant if *p* value >0.05

VAS scores are represents in Table 3, it reveals that immediate after operation, all groups had comparable pain. The overall mean VAS score among the Cold pack gel group was 4.216 ± 0.716, Gum chewing group was 4.314 ± 0.908, compared to control group was 5.714 ± 1.232 with statistically significance differences among groups.

**Table 3:** Comparison pain mean score between three groups

	Gum chewing (*N*=53)	Cold pack gel (*N*=53)	Control group (*N*=53)	*Kruskal Wallis* *P*.value
0 hr Mean±S.D	3.12± 1.1	3.47± 0.9	3.35± 0.87	0.812 >0.05
4 hr Mean±S.D	4.21± 0.87	4.11± 0.74	5.89± 1.2	3.886 <0.05*
8 hr Mean±S.D	4.76± 1.10	4.45± 0.62	6.33± 1.5	5.661 <0.01**
12 hr Mean±S.D	5.36± 0.84	5.15± 0.73	7.60± 1.6	5.800 <0.01**
24 hr Mean±S.D	4.12± 0.63	3.90± 0.59	5.40± 0.99	3.908 <0.05*
Overall mean score of pain	4.314±0.908	4.216±0.716	5.714±1.232	4.0134 <0.05*

Highly significant if *p* value <0.01

Significant if *p* value <0.05

Insignificant if *p* value >0.05

**Fig. 2:**
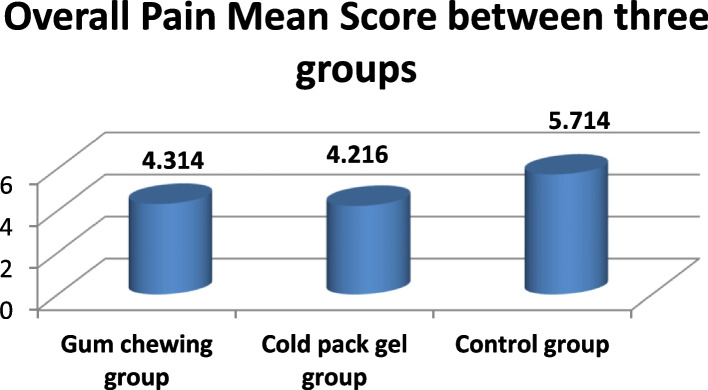
Overall mean score of pain between three groups

Mean VAS scores are displays in Fig. 2, it shows that both cold pack gel and gum chewing reduce post-C/S pain in women. While cold pack gel proved more effective than gum chewing compared to control group.

Table 4 indicates that there was a significant difference between the study groups at *p* value < 0.05* in the management of pain following cesarean section within the first 24 h. The current study also showed that, compared to the control group which consisted of just 7.5% of participants, over one quarter in cold therapy group, less than one quarter in Gum chewing group (32.1 and 28.3%, respectively) stopped taking analgesics 24 h after surgery.

**Table 4:** Management of post cesarean section pain in the first 24 h

Items	Gum chewing (*N*=53)		Cold pack gel (*N*=53)		Control group (*N*=53)		*Chi-square* *p*. value
	N	%	N	%	N	%	
Diclofenac only	23	43.4	27	50.9	16	30.2	4.023 <0.05*
Pethidine only	8	15.1	4	7.5	16	30.2	
Intrathecal morphine only	5	9.4	3	5.7	8	15.1	
Multiple analgesics	2	3.8	2	3.8	9	16.9	
No analgesics	15	28.3	17	32.1	4	7.5	

Highly significant if *p* value <0.01

Significant if *p* value <0.05

Insignificant if *p* value >0.05

## Discussion

The percentage of births resulting in a Caesarean section (CS) has significantly increased in recent years, making up 15% to 25% of all births. Moderate to severe pain after the operation is a commonly reported issue during the post-CS period. Predicting the level of postoperative pain would be ideal for tailoring analgesia [23]. Thus, the current study sought to evaluate the effect of gum chewing and cold therapy on postoperative cesarean women’s self-assessed pain levels and narcotics use.

The women under study were chosen by the researchers and then randomly assigned to three groups, ensuring that all three groups had comparable and matching characteristics. In the postoperative obstetric ward, our study found that both cold pack gel and gum chewing reduce post-C/S pain in women at 4 h, 8 h, 12 h, and 24 h, with no impact at 0 h. While cold pack gel proved crucial and far more effective than gum chewing compared to control group.

One possible explanation for this could be that pain perception mostly travels through small fibers, while sensations of cold, touch, or pressure are sent through large fibers with a faster conduction velocity. The gate would close when a big non-nociceptive fiber in a patient was stimulated simultaneously with pain by cold or pressure. Both nociceptive and non-nociceptive neurons exhibited presynaptic inhibition according to [24].

These results supported by [24] who observed statistically significant pain reduction with cold pack use from 6 to 24 h postoperatively, which is consistent with these results. Additionally, a study by [25] that used a low transverse incision and cold compression for 6 h following a cesarean section showed a significant decrease in discomfort from 6 to 12 h after surgery. Moreover, these findings are consistent with a study conducted in 2017 by [23], which found that the average pain scores during rest and movement were 0.40 ± 0.013 and 0.83 ± 0.017 (VAS score), respectively, 48 h after surgery.

Additionally, Gum-Chewing following two hours of Cesarean minimized the pain score in the first 24 h, according to [26]. Furthermore, [27] documented that the nursing intervention program for women undergoing cesarean sections was successful in enhancing the psychological well-being of the women and mitigating their postoperative discomfort. In another study, [28] and [21] found that cryotherapy could only relieve post-gynecologic surgery discomfort for six and twelve hours, respectively.

Similarly, a study conducted in 2017 by [29] who assessed postoperative pain in women having cesarean sections and found that this group experiences high intensity postoperative pain, highlighting the significance of pain assessment for the implementation of curative and preventive measures to minimize losses in women's recovery. Additionally, [30] found that cold therapy helped patients who had low midline incisions for general surgery have less pain after surgery.

According to the results of the current study, both cold pack gel and gum chewing reduce dependence on narcotic use during the first 24 h following C/S. The results of this study also showed that, in comparison to a very tiny percentage in the control group, more than one quarter cold therapy group, less than one quarter Gum chewing group. This might be because women who have planned cesarean sections use fewer opioids because they experience less pain. This also indicates that, both cold pack gel and gum chewing were effective in reduce post-C/S pain.

These findings, In the same line according to [25], showed that the use of cold pack gel decreased patients' pain scores six and twelve hours following surgery, as well as their intake of narcotic medications. Additionally, gum chewing helps patients who have scheduled cesarean sections take less opioids and report less pain [31]. Furthermore, [26] found that chewing gum is an easy-to-use technique that can successfully speed up healing following cesarean delivery. Chewing gum has been linked to fewer discomforts and complication rates, according to [32].

Moreover, [33] reported that the adjunctive cold gel pack was effective in lowering postoperative pain six hours following cesarean birth without raising any safety issues and lowering the total amount of opioids used. Furthermore, [24] found that cryotherapy could decrease the total need for opioids and reduce postoperative discomfort from six hours to within twenty-four hours of the procedure. Additionally, [34] found that employing 24-h ice pack compression reduces postoperative pain and morphine use following surgery till postoperative day 3.

## Conclusion

Cold pack gel and gum chewing offer simple and cost-effective alternatives for postoperative pain management; however, cold pack gel has been shown to be substantially more effective in improving pain scores at four, eight, twelve, and twenty-four hours after surgery. In addition to its impact on reducing the need for opioid prescription drugs.

### Limitation of the study

The present research is limited by the incapacity to blind both the participants and the attending nurses about who was given normal postoperative care alone or an additional cold gel pack or gum chewing. Consequently, bias may have been present during the VAS score evaluation process. Additionally, the individuals were motivated and interested in both gum chewing and the study. Because of this, the results might not apply to the general population without any incentives.

Moreover, Although the study focuses on the Port Said area, addressing a research gap in this region, the results may not be applicable to all locations or populations in general. This is because the study may have been conducted on a limited sample or in a specific environment that may not reflect the reality in other locations or population groups.

### Recommendation

The following recommendations were made in light of the findings of the existing studies:

• After a cesarean delivery, nurses should assess the comfort measures offered and counsel women on using non-opioid pain management techniques.

• Cold pack gel and gum chewing should be advised as regular, alternative therapy for pain control in women who have had C/S.

• Introducing health education programs that inform expectant mothers about the advantages of alternative therapy, such as chewing gum and cold pack gel, drinking plenty of water early in the day, and walking after a cesarean section.

### Further research

To confirm the impact of a standardized chewing gum intervention program and cold pack gel (start time, frequency, chewing dose, duration, etc.) after C/S, we can increase the sample number and conduct excellent randomized controlled trials.

## Data Availability

Due to confidentiality concerns, the data used in the current study cannot be made publicly available. However, they are available from the corresponding author upon reasonable request.
